# Imaginator

**DOI:** 10.1192/bjo.2024.662

**Published:** 2024-08-01

**Authors:** Emily Gardner-Bougaard, Athina Sevri, Martina Do Simplicio

**Affiliations:** 1 London, United Kingdom; 2 Imperial, London, United Kingdom

## Abstract

**Aims:**

Self-harm affects around 20% of all young people in the UK. Treatment options for self-harm remain limited and those available are either non-specific or long and costly and may not suit all young people. There is an urgent need to develop new scalable interventions to address this gap.

Imaginator is a novel imagery-based intervention targeting self-harm initially developed for 16–25-year-olds. It is a blended digital intervention delivering Functional Imagery Training (FIT) via therapist sessions and a smartphone app. In this study we piloted a new version of Imaginator extended to adolescents from age 12 after co-producing a new app with a diverse group of young people experts-by-experience.

We aimed to assess feasibility of delivering Imaginator in Children and Adolescent Mental Health Services (CAMHS) and adult secondary mental health services and gather young people's feedback on the intervention

**Methods:**

Participants were recruited from West London NHS Trust Tier 2 CAMHS and adult Mental health Integrated Network Teams (MINT) teams. They underwent a baseline screening and were allocated to a therapist for three face-to-face FIT sessions in which the app was introduced followed by five phone support sessions. Outcome assessments were conducted after completing therapy, approximately 3-months post-baseline, including questionnaire measures and a qualitative feedback interview.

Qualitative data were analysed using a co-produced thematic analysis method with lived experience co-researchers.

**Results:**

Thirty-four participants were referred (31 female, 2 male, 1 transgender; mean age = 18.4), of which 30 met inclusion criteria and completed screening. Out of 25 who started therapy 16 completed the intervention. Only 15 completed the quantitative outcome assessment, and 10 the interviews. There was an overall reduction in number of self-harm episodes over 3-months from pre- to post-intervention

Five main themes were identified: Imaginator therapy impact, mental imagery acceptability and efficacy, usefulness and usability of the app, integration of the app in therapy and need for improvements. Young people found Imaginator helpful at improving their mental health, in particular the use of mental imagery techniques. The app was overall well received but improvements were suggested.

**Conclusion:**

Our study suggests that Imaginator can be extended to adolescents, is acceptable and has potential as a brief intervention reducing self-harm in young people under mental health services. A future RCT is needed to robustly test the intervention efficacy, after considering issues around high attrition in outcome measures.

